# Cuprous oxide-based nanocrystals with combined chemo/chemodynamic therapy to increase tumor drug sensitivity by reducing mitochondria-derived adenosine-triphosphate

**DOI:** 10.1080/10717544.2022.2121450

**Published:** 2022-09-25

**Authors:** Haoran He, Jiaming Wu, Min Liang, Yao Xiao, Xuejian Wei, Yuqin Cao, Zhiheng Chen, Tian Lin, Miaosheng Ye

**Affiliations:** a The Second People’s Hospital of Nanhai District, Guangdong Provincial People’s Hosptial’s Nanhai Hospital, Foshan, Guangdong Province, China; b Department of Gastrointestinal Surgery, The First Affiliated Hospital of Jiaxing University, Jiaxing, Zhejiang Province, China; c Department of Oncology, Innovation Centre for Advanced Interdisciplinary Medicine, Guangzhou Key Laboratory of Enhanced Recovery after Abdominal Surgery, The Fifth Affiliated Hospital of Guangzhou Medical University, Guangzhou Medical University, Guangzhou, Guangdong Province, China; d Nanfang Hospital, The First School of Clinical Medicine, Southern Medical University, Guangzhou, Guangdong Province, China

**Keywords:** Cuprous oxide (Cu_2_O), drug resistance, P-glycoprotein (P-gp), chemodynamic therapy (CDT), mitochondria damage

## Abstract

Gastrointestinal (GI) tumor is a serious disease with high mortality rates and morbidity rates worldwide. Chemotherapy is a key treatment for GI, however, systematic side effects and inevitable drug resistance complicate the situation. In the process of therapy, P-glycoprotein (P-gp) could remove chemotherapy drugs from cells, thus causing multi-drug resistance. Chemodynamic therapy (CDT) utilizing Fenton chemistry has been used for cancer therapy, along with various combination therapies. The reactive oxygen species produced by CDT could inhibit P-gp’s efflux pump function, which reduce chemoagents excretion and reverse drug resistance. In the present study, we developed novel nanocrystals (Cu_2_O@Pt NCs) to overcome drug resistance by reducing mitochondria-derived ATP through chemo/CDT in GI cancer. Furthermore, *in vivo* results in tumor-bearing mice demonstrated that treatment with Cu_2_O@Pt NCs with CDT and chemotherapy could achieve the most effective antitumor therapeutic effect with the least amounts of adverse effects. As a result, Cu_2_O@Pt NCs could provide a promising strategy for chemo/CDT-synergistic therapy.

## 1. Introduction

Gastrointestinal (GI) tumor is one of the five most common cancer types in the world and the major contributor to cancer-related death worldwide (Bray et al., 2018; Sung et al., 2021). To date, surgery remains the mainstay of GI tumors treatment, and it is a trend to shift to minimally invasive surgery (Akahoshi et al., 2018). Meanwhile, given either preoperatively or postoperatively, adjuvant chemotherapy could improve survival compared with surgery alone because 80% of patients with GI cancer are diagnosed in the advanced stage (Venerito et al., 2016; Chen et al., 2021). Although the optimum chemotherapy regiment is the traditional platinum (Pt)-based chemotherapy, the effectiveness is dramatically compromised owing to the drug resistance and disease recurrence developed in patients with advanced GI (Rottenberg et al., 2021; Behranvand et al., 2022). Therefore, it is vital to discover more effective chemotherapeutics to enhance the efficiency of Pt while reducing side effects.

Effective drug delivery systems (DDSs) based on nanosized technologies have been proposed recently as a useful strategy for overcoming these issues (Heng, 2018; Unsoy & Gunduz, 2018; Jain, 2020; Li et al., 2021). Because of their high drug loading capacity, these nanoparticles can efficiently penetrate cancer sites due to their high permeability and retention (Adepu & Ramakrishna, 2021; Zhang et al., 2022). However, despite the enhanced absorption by smart nano-DDSs, P-glycoprotein (P-gp) can still pump intracellular chemotherapy drugs out of cells, causing a reduction in drug concentration inside of cells and compromising chemotherapy efficacy, inducing multidrug resistance (MDR) (Mollazadeh et al., 2018; Waghray & Zhang, 2018). Therefore, blocking the efflux pump function of P-gp directly would be an effective strategy for treating MDR. Recent research has shown that chemotherapy combined with chemodynamic therapy (CDT) is an effective way against MDR (Mollazadeh et al., 2018; Waghray & Zhang, 2018), since CDT can impair the function of P-gp. Thus, MDR cancer cells showed a resurgence of drug sensitivity, namely, the enhanced absorption of cancer drugs caused more apoptosis to occur nearby the cancer cells. Cu_2_O nanoparticles have excellent CDT properties and are reliable drug delivery agents (Chang et al., 2020). In a consistent CDT setting, nano-DDS derived from Cu_2_O could suppress the efflux pump function of P-gp, providing a more effective therapy. The nano-DDS derived from Cu_2_O could directly suppress P-gp’s efflux pump function to achieve a better therapeutic effect, when applied with consistent CDT.

In the present study, we developed CDT and chemotherapy by novel nanocrystals (Cu_2_O@Pt NCs). The released Cu ions from the Cu_2_O@Pt NCs could act as Fenton catalyst to boost the generation of reactive oxygen species (ROS) and what’s more, under the synergistic effect of Pt, the generation of ROS would increase further. Because of the oxidative stress induced by CDT, the Cu_2_O@Pt NCs were capable of elevating ROS levels and eliciting mitochondrial dysfunction. What’s more, P-gp’s function could be inhibited by CDT, which reduce cisplatin excretion and reverse drug resistance. *In vivo* results in tumor-bearing mice demonstrated that treatment with Cu_2_O@Pt NCs with CDT and chemotherapy could achieve the most effective antitumor therapeutic effect with the least amounts of adverse effects.

## 2. Experimental section

### 2.1. Materials

Copper chloride dihydrate (CuCl_2_·2H_2_O), polyvinylpyrrolidone (PVP K30, MW = 40 000 Da), sodium hydroxide (NaOH), and ascorbic acid (AA) were purchased from Sinopharm Chemical Reagent Co., Ltd. (Shanghai, China). Cisplatin was obtained from Solarbio (Shanghai, China). Mercapto-polyethylene glycol (SH-PEG, MW = 5000) was acquired from Shanghai Advanced Vehicle Technology (Shanghai, China). Deionized water (DI water, 18.2 MΩ cm) obtained from a Milli-Q water system was used for all experiments. All of the chemicals and reagents were of analytical quality and were utilized without further purification.

### 2.2. Synthesis of Cu_2_O@Pt-PEG nanocrystals

Briefly, the Cu_2_O NCs were made by mixing AA with aqueous Cu^2+^ and NaOH and capping them with PVP. Under magnetic stirring, PVP (600 mg) and CuCl_2_·2H_2_O (0.5 mmol) were dissolved in DI water (20 mL), followed by the NaOH aqueous solution (8.0 mmol). In addition, AA (1.0 mmol) was added and then stirred for 20 min. Orange Cu_2_O NCs developed almost immediately with the addition of AA. Centrifugation was used to collect the Cu_2_O NPs, which were then extensively cleaned with DI water and ethanol multiple times.

Second, for modification of PEG, the Cu_2_O NCs (1 mg) and SH-PEG (10 mg) were disseminated in 20 mL DI water under stirring at 60 °C for 24 h. The Cu_2_O-PEG NPs were then purified by centrifugation (at 12,000 rpm for 8 min) and kept at 4 °C until needed.

Finally, for the synthesis of Cu_2_O@Pt-PEG, Cu_2_O-PEG NCs (1 mg) and cisplatin (1 mg) were combined and agitated for 24 h in ethanol (5 mL). The ethanol was used to wash the precipitate three times. Cu_2_O@Pt-PEG was the name given to the final composite.

### 2.3. Characterization of Cu_2_O@Pt-PEG nanocrystals

A UV-5500PC UV–vis spectrophotometer (Shanghai METASH, China) was used to record UV–vis absorbance spectra. Transmission electron microscope (TEM) images were captured using a JEM-1400F TEM (JOEL) operating at 200 kV. Inductively coupled plasma atomic emission spectrometry (ICP-AES) and atomic absorption spectroscopy (AAS) were used to determine the metal content of the samples using a Prodigy instrument and a HITACHI Z-2300 instrument, respectively. Zeta PALS system (Nanobrook Zeta PALS, Brookhaven Instruments Corporation, USA) was employed to measure the hydrodynamic diameter and zeta.

### 2.4. Intracellular uptake assay

At 37 °C and 5% CO_2_, mouse gastric cancer cell (MFC) cells were cultured with 10% fetal bovine serum (FBS) and the rhodamine (Rho)-labeled Cu_2_O@Pt-PEG NCs was added. After incubation, confocal laser scanning microscope (CLSM) was used to observe the cells from 1 to 24 h, and endocytosis of Cu_2_O@Pt-PEG NCs was also observed by CLSM.

### 2.5. *In vitro* anti-tumor assay

MFC were cultured with 10% fetal bovine serum (FBS) and then treated with Cu_2_O-PEG NCs or Cu_2_O@Pt-PEG NCs for 48 h. The cells were then analyzed using the Live&death assays (Sigma-Aldrich, USA), cell counting kit-8 (CCK-8), and lactate dehydrogenase (LDH) in accordance with the manufacturer’s guidelines.

### 2.6. Transwell assays

Transwell plates (BD Biosciences) were used for transwell experiments. A total of 5 × 10^4^ cells and 1% FBS were added to each of the upper compartments according to the manufacturer’s instructions. The lower compartments were induced with 20% FBS. Afterward, Cu_2_O-PEG NCs was added to the lower chambers. Taking out the upper compartment after 24 h of incubation at 37 °C and fixing the cells in methanol for 30 min was the next step, then the upper surface cells of the upper compartment were removed with cotton swabs. Cells invading to the lower side of the upper compartment were stained with 1% crystal violet.

### 2.7. Measurement of mitochondria damage

Cultured in DMEM with 10% FBS, MFC were seeded and grown for 24 h. Then, the cells were treated with culture medium (set as the blank group) or Cu_2_O-PEG NCs for 24 h. To explore anti-tumor mechanism, aforementioned treated cells were also analyzed with ROS (Sigma-Aldrich), Rho123 assay, mitochondrial ROS (mitoROS; AAT Bioquest, Wuhan, China), mitochondrial permeablity transition pore (MPTP) kit (BestBio, Shanghai, China), JC-1 kit (Keygen, Nanjing, China), and mitochondrial complex I–V (Solarbio, Beijing, China).

### 2.8. *In vivo* anti-tumor study

To construct a tumor-bearing BALB/c mouse model, harvested MFC were suspended in a suitable amount of PBS. The mice were then injected with 50 mL of suspension in their left flank. When the tumor volume reached 50 mm^3^, six groups of mice were divided, which were treated with saline (negative control), Cisplatin (2 mg/kg, Low doge), Cisplatin (5 mg/kg, high doge), and Cu_2_O-PEG NCs or Cu_2_O@Pt-PEG NCs solutions via tail vein injection every 2 days. The tumor volumes were recorded daily for 14 days.

#### 2.8.1. In vivo biosafety and metabolism study

Cu_2_O@Pt-PEG NCs were intravenously injected separately into the healthy BALB/c mice for investigating the in vivo Biosafety and Metabolism of Cu_2_O@Pt-PEG NCs. A digestion solution containing digesting aqua regia was used at the indicated time points (0 h, 6 h, 12 h, 24 h) for collecting, weighing, and dissolving the main organs and tumor. The concentrations of Cu ions in different samples were detected by ICP-MS. Meanwhile, main organs (heart, liver, spleen, lung, and kidney) were obtained for H&E staining.

### 2.9. Statistical analysis

Results are expressed as mean ± standard deviation (SD). Except where indicated otherwise, all tests have been repeated three times and produced comparable results. Unpaired *t* tests were used to examine statistical data, analysis of variance, and Student’s *t* tests (ANOVA). At *p* < .05, differences were judged statistically significant.

## 3. Results and discussion

### 3.1. Synthesis of Cu_2_O@Pt-PEG nanocrystals

**Figure 1.**
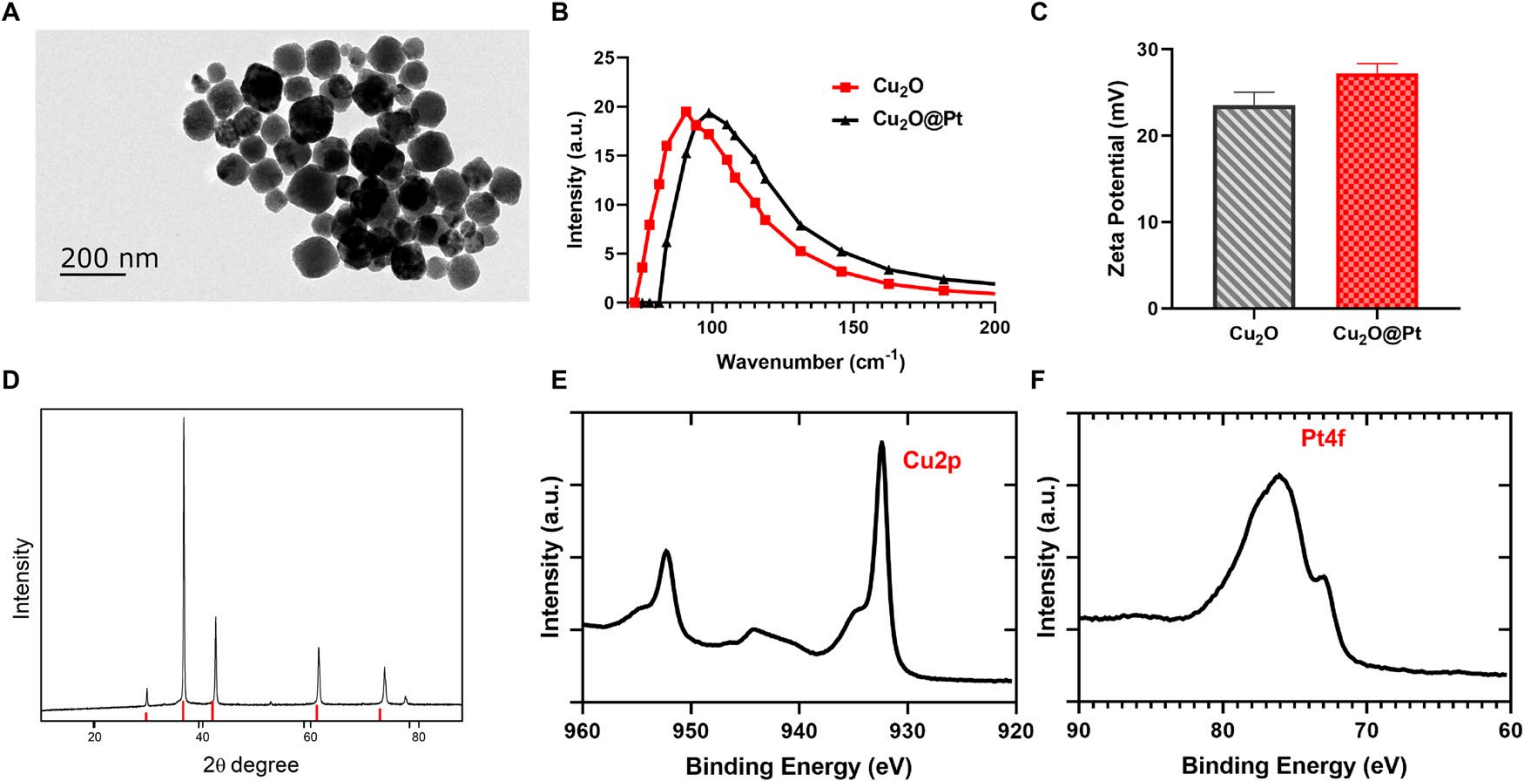
A: TEM images Cu_2_O NCs; (B) DLS of Cu_2_O NCs and Cu_2_O@Pt NCs. C: Zeta potential of Cu_2_O NCs and Cu_2_O@Pt NCs. D: XRD analysis of Cu_2_O@Pt NCs; (E) X-ray photoelectron spectroscopy of Cu_2_O@Pt NCs, the element of Cu and Pt.

Figure 1(A) shows a cubic morphology with an average diameter of 75 nm for Cu_2_O by the TEM. As shown in Figure 1(B), dynamic light scattering (DLS) measurement found that the hydrodynamic size (Dh) of the Cu_2_O and Cu_2_O@Pt NCs were respectively 84.41 ± 2.01 nm and 102.32 ± 2.34 nm. Results from zeta potential showed 24.31 ± 2.00 mV and 29.46 ± 1.52 mV for Cu_2_O NCs and Cu_2_O@Pt NCs, indicating the feasibility for biomedical applications (Figure 1(C)). What’s more, powder X-ray diffraction (XRD) analysis was used to establish the chemical composition and phase structure of Cu_2_O; every reflection peaks were compatible with cubic Cu_2_O (Chang et al., 2020) (Figure 1(D)). Meanwhile, X-ray photoelectron spectroscopy (XPS) confirmed that Cu and Pt were the main elements inside Cu_2_O@Pt, suggesting the NCs were pure (Figure 1(E,F)). The content of Pt and Cu, determined by inductively coupled plasma mass spectrometry, was 3.5% and 24.5%, respectively.

### 3.2. Characterization of Cu_2_O@Pt-PEG nanocrystals

**Figure 2.**
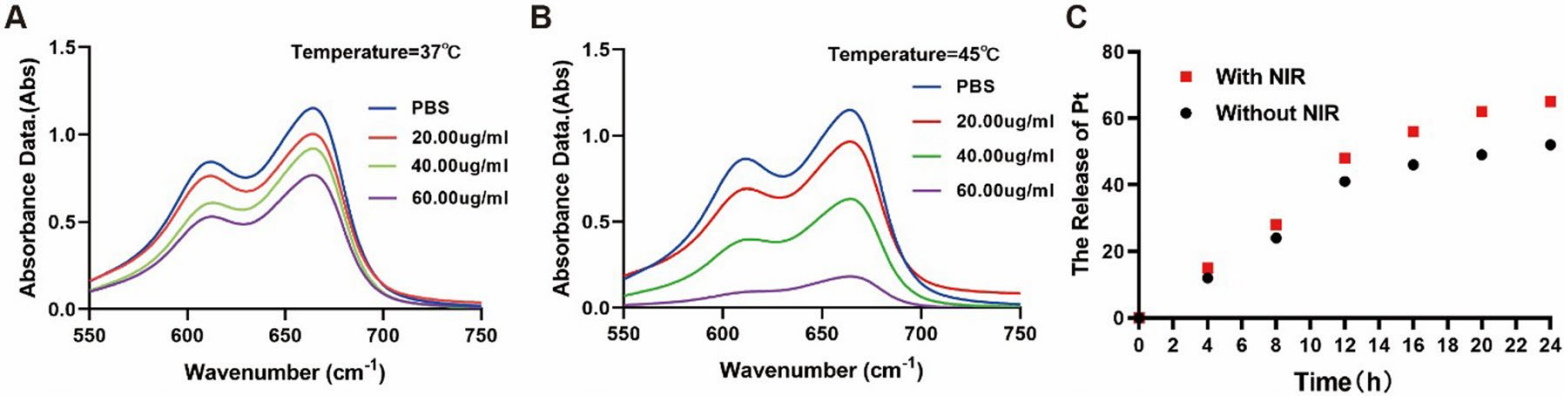
Fenton-like properties assessed by MB in the presence of Cu_2_O NCs at different concentrations at (A) 37 °C and (B) 50 °C; (C) time-dependent Pt release from the Cu_2_O@Pt NCs with and without NIR.

To determine whether Cu_2_O@Pt-PEG produce Fenton-like effect, MB assays based on MB degradation were used (Wu et al., 2020). In the presence of high concentrations of Cu_2_O@Pt-PEG, MB absorption decreased, indicating abundant **·**OH production by Cu_2_O@Pt-PEG NCs (Figure 2(A)). In accordance with previous reports, the results were positive (Chang et al., 2020; Gao et al., 2022). Moreover, to investigate the role of temperature in the Fenton-like reactions of the Cu_2_O@Pt-PEG NCs, we placed the reaction systems in a water bath at 50 °C. In a water bath at 50 °C, the Cu_2_O@Pt-PEG NPs had a lower MB absorption than those at 37 °C (Figure 2(B)**)**. The Cu_2_O@Pt-PEG NPs solution (120 mg/mL) demonstrated a 10-fold enhancement at 50 °C when compared to those at 25 °C. The results showed that increased temperature could significantly improve the Fenton-like effect of Cu_2_O@Pt-PEG NPs, suggesting a more effective method of CDT by mild photothermal treatment. To detect the ability to release Pt, the boosted Pt from Cu_2_O@Pt-PEG nanoparticles was assessed by ICP-AES. As shown in Figure 2(C), more than 50% of the Pt was produced within 24 h. Meanwhile, Cu_2_O@Pt-PEG NPs released more Pt with NIR, resulting in more effective chemotherapy effects. What’s more, to simulate acidic tumor microenvironment, we conducted the supplement experiment to explore the release studies of Pt and Cu in the PBS and in a solution with low pH. As shown in the Supplementary Figure S1, Cu_2_O@Pt-PEG NPs released more Pt with low pH (Supplementary Figure S1(A)) and the experiment for Cu had similar results (Supplementary Figure S1(B)). As a result, Pt could better diffuse from the Cu_2_O@Pt NCs due to thermodynamic effects, which are consistent with the previous study.

### 3.3. *In Vitro* cellular uptake and anti-tumor effect of Cu_2_O@Pt-PEG nanocrystals

Platinum (Pt) including cisplatin, oxaliplatin, and carboplatin, which refers to Pt(II) drug, have been preferred as first-line agents for the chemotherapy of patients with GI tumor (Johnstone et al., 2013; Chau et al., 2016; Dilruba & Kalayda, 2016; Yang et al., 2020). Chemotherapy mainly induces caspase-dependent apoptosis within cancer cells, while long-term exposure leads to enhanced anti-apoptosis signal or decreased drug sensitivity (Carneiro & El-Deiry, 2020). However, it is very difficult for intravenously injected chemo-agents to selectively enter tumor region during the blood circulation process (Bregni et al., 2020). To achieve better therapeutic effects, clinicians often choose to using high dose of drugs for treatment, while this regimen is prone to cause systemic side effects, which may lead to the failure to tolerate and discontinue treatment for patients with cancer.

**Figure 3.**
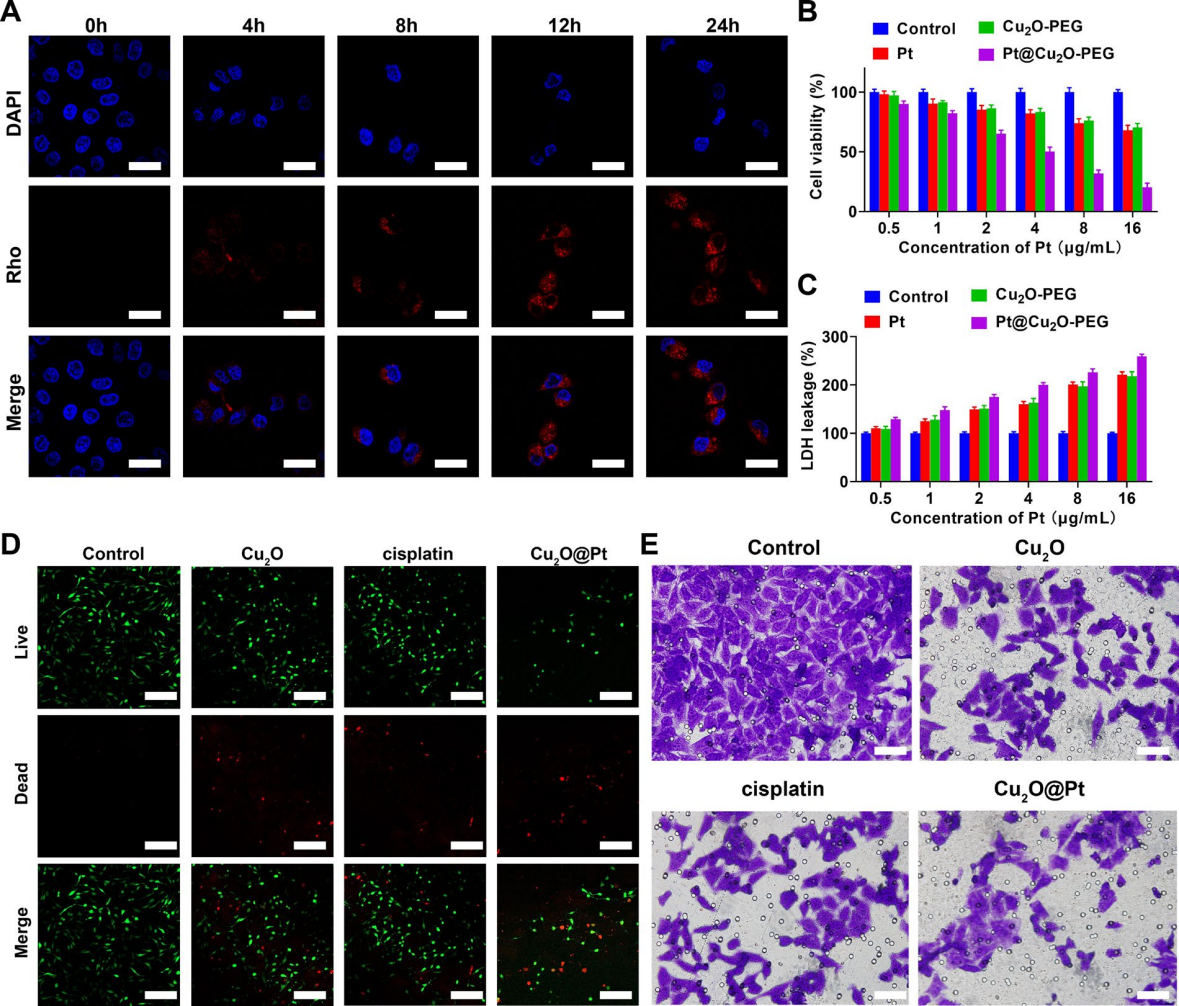
A: CLSM images of MFC cells treated with Cu_2_O@Pt-PEG NCs for 1–8 h. Scale bar: 20 μm. B: CCK-8 assay of MFC cells after treated with Cu_2_O-PEG and Cu_2_O@Pt-PEG. C: LDH leakage assay of MFC cells after treated Cu_2_O-PEG and Cu_2_O@Pt-PEG. D: Live&Dead images of MFC cells after treatment. Scale bar: 100 μm. E: Transwell assay of MFC cells after treated Cu_2_O-PEG and Cu_2_O@Pt-PEG.

Therefore, it is a very important issue for efficient drug delivery during GI cancer therapy. Inspired by the fact that nanosized DDSs have evolved to fully adapt to tumor surroundings owing to enhanced permeability and retention (EPR) effect (Kobayashi et al., 2013; Goos et al., 2020), Cu_2_O-PEG nanocarriers were applied for the delivery of Pt. To verify that, the cellular uptake Cu_2_O@Pt-PEG NCs were performed using CLSM, which were pre-labeled with rhodamine (Rho). From CLSM images, it could be observed that the cellular uptake of Cu_2_O@Pt-PEG NCs was at a time-dependent manner. Especially, visible red fluorescence in cancer cells could be significantly observed only after 4 h of incubation. Cancer cells showed the stronger red fluorescence after 8 h of incubation and only when the incubation time reached 12 h, did the accumulated Rho fluorescence in the cytoplasm eventually reach a very high level, which could be obviously understood by corresponding quantitative analysis. In particular, we could find no significant increase of Rho fluorescence at the incubation time of 24 h, compared to 12 h, indicating that 12 h was the highest cellular uptake time (Figure 3(A), Supplementary Figure S2). Given the excellent cellular uptake and Fenton-like catalytic activities of Cu_2_O@Pt-PEG, we then performed CCK-8 and LDH assay to evaluate their anti-tumor effect. From CCK-8 results, cell viability rate of cancer cells declined for all groups at dose-dependent manner, but at a totally different slope. When the concentration of Pt was 16 μg/mL, Pt (∼30%) and Cu_2_O-PEG (∼30%) showed more significant inhibitory effect to cancer cells. Interestingly, Cu_2_O@Pt-PEG (refer to chemo/CDT) caused twofold changes higher cell toxicity (∼80%) than Pt and Cu_2_O-PEG (Figure 3(B)**)**. Consistently, LDH as a cell damage assay showed that with increasing concentration, LDH decreased in Pt, Cu_2_O-PEG, and Cu_2_O@Pt-PEG treated groups, whereas the LDH rate was more evident in the Cu_2_O@Pt-PEG group (Figure 3(C)**)**. Therefore, CCK-8 and LDH assay indicated that synthetic chemo/CDT effects of Cu_2_O@Pt-PEG NCs showed a much better results than those single chemotherapy (Pt) or single CDT effect (Cu_2_O-PEG).

To detect the chemo/CDT-therapeutic effectiveness, incubation of cancer cells with Cu_2_O@Pt-PEG (8 μg/mL of Pt) was managed for further experiments. Compared with cell-impermeable Propidium Iodide (PI) that could merely stain the dead cells’ nuclei with strong red fluorescence, low toxic calcein-AM could penetrate a living cell membrane, while being hydrolyzed into calcein and brightening green fluorescence (Uggeri et al., 2004). As shown in live&death results, Pt, Cu_2_O-PEG, and Cu_2_O@Pt-PEG treatments all demonstrated reduced green intensity, accompanied with increased red intensity (Figure 3(D), Supplementary Figure S3). Furthermore, Transwell assay results revealed that significantly Pt, Cu_2_O-PEG, and Cu_2_O@Pt-PEG attenuated the migratory ability of cancer cells compared with control group (Figure 3(E), Supplementary Figure S4). To date, Cu_2_O@Pt-PEG did showed most decreased green intensity and upregulated red intensity in live&death research, and most suppressed migrated cell numbers in Transwell experiments. The aforementioned results suggested Cu_2_O@Pt-PEG NPs not only possesses great toxicity, but also to bust anti-proliferation and anti-metastasis effect to cancer cells.

**Figure 4.**
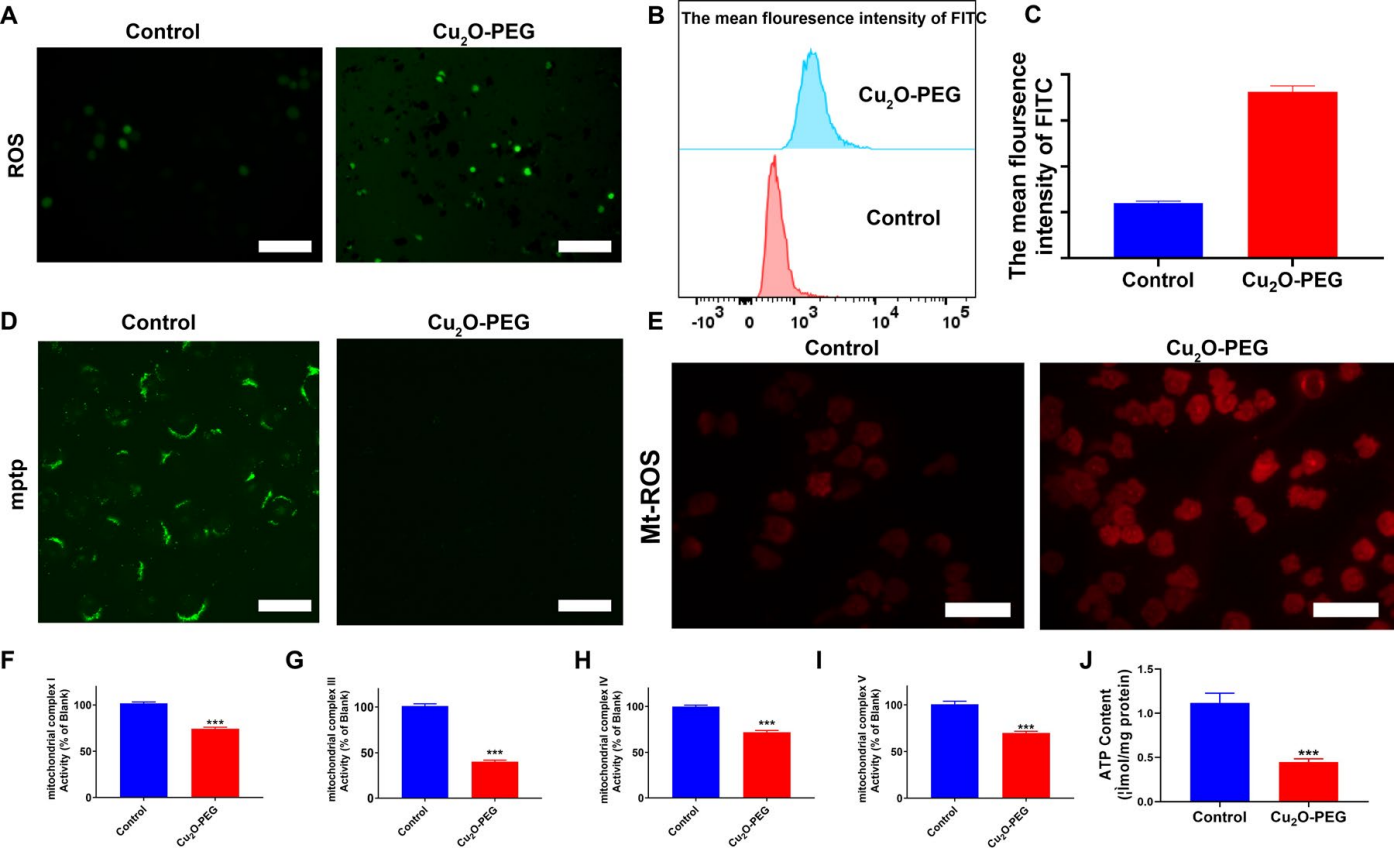
A: Total ROS image of MFC cells after treated with PBS (Control) and Cu_2_O-PEG. Scale bar: 100 μm. B and C: Quantified plot of Rho content in MFC cells, detected by flow cytometry; (D) MPTP images of MFC cells after treatment with PBS (Control) and Cu_2_O-PEG. Scale bar: 20 μm. E: Total Mt-ROS image of MFC cells after treated with PBS (Control) and Cu_2_O-PEG. Scale bar: 50 μm. F–I: The activities of complexes I/III/IV/V of MFC cells after treated with Cu_2_O-PEG. J: ATP production of MFC cells after treated with Cu_2_O-PEG.

### 3.4. CDT effect induced by Cu_2_O@Pt-PEG could reverse drug resistance by decreasing mitochondria-derived ATP

Though the excellent anti-tumor effect of Cu_2_O@Pt-PEG NCs has been proved, the mechanism is still unclear. In spite of smart DDSs enhancing absorption, P-glycoprotein (P-gp) has been shown to pump out chemoagents endocytosed by cancer cells (Mollazadeh et al., 2018). ATP-binding cassette (ABC) transporters such as P-gp, which have a molecular mass of 170 kD, act as ‘drug pumps’ that consume ATP (Leopoldo et al., 2018). In general, the efflux of intracellular drugs via energy-dependent P-gp causes the concentration of drugs inside the cell to decrease and the development of resistance to those drugs (Waghray & Zhang, 2018). Accordingly, reducing ATP supplements could be a promising strategy for suppressing P-gp’s efflux pump function and the reversal of drug delivery. In addition, the complex enzymatic activities of the mitochondrial respiratory chain determine intracellular ATP production. It has been demonstrated that the inevitable product of cellular metabolism, ROS, can cause mitochondrial damage (Yang et al., 2019), whereas the CDT effect can increase ROS levels inside the cells (Tang et al., 2019). According to that, we assumed that these Cu_2_O@Pt-PEG NCs might stimulate the intracellular generation of ROS and trigger mitochondrial damage via its CDT effect. Consequently, both ATP synthesis and the efflux pump ability of P-gp might be dysfunctional due to the insufficient energy supplement.

To confirm this, we treated cancer cells with our designed Cu_2_O-PEG NCs first and detected their ROS generation ability. After the intervention, ROS generation in Cu_2_O-PEG-treated cells was remarkably increased, which was exhibited via DCFH-DA, a biological probe to detect the intracellular levels of ROS (Figure 4(A), Supplementary Figure S5). To further explore the ability of retention of intracellular drugs, the cancer cells were treated with our designed Cu_2_O-PEG NCs first and detected the ability of Rho123 retention, which showed the ability of drug retention in the cells. After the intervention, the fluorescence of Rho123 in Cu_2_O@Pt-PEG-treated cells was remarkably increased, indicating the dysfunctional efflux pump of P-gp (Figure 4(B), Supplementary Figure S6). Apart from this, the matrix metalloproteinase (MMP) were detected by JC-1 kit and the group with Cu_2_O-PEG NCs collapsed sharply **(**Figure 4(C), Supplementary Figure S7). The results also revealed that Cu_2_O-PEG NCs further enhanced the opening of mPTP, with a sharp decrease in microsomal triglyceride transfer protein (MTP) (Figure 4(D), Supplementary Figure S8). Therefore, our results elucidate that the CDT effect induced by Cu_2_O-PEG NPs could destroy mitochondria. Subsequently, MitoSOX Red was applied to discern superoxide as an indicator in mitochondria using CLSM analysis, to illuminate whether the generated ROS could cause inner mitochondrial damage. Notably, red fluorescence (mitochondrial ROS, also called mitoROS) was rapidly increased in the Cu_2_O-PEG-treated group (Figure 4(E), Supplementary Figure S9). Moreover, we found that Cu_2_O-PEG NPs also reduced the activities of complexes I/III/IV/V in treated cancer cells, indicating Cu_2_O-PEG NPs generated ROS injured NADH-dependent Electron transport chain (ETC). The procedure of ATP generation was evidently suppressed because of the damage to mitochondria complexes (Figure 4(F–I)). Subsequently, more Rho123 was sequestered, indicating the dysfunctional efflux pump of P-gp. These results indicated mitochondrial-complexes I/III/IV/V chain in Cu_2_O-PEG NPs-treated cells was damaged, resulting in ATP production decline and dysfunction of P-gp pumps (Figure 4(J)).

**Figure 5.**
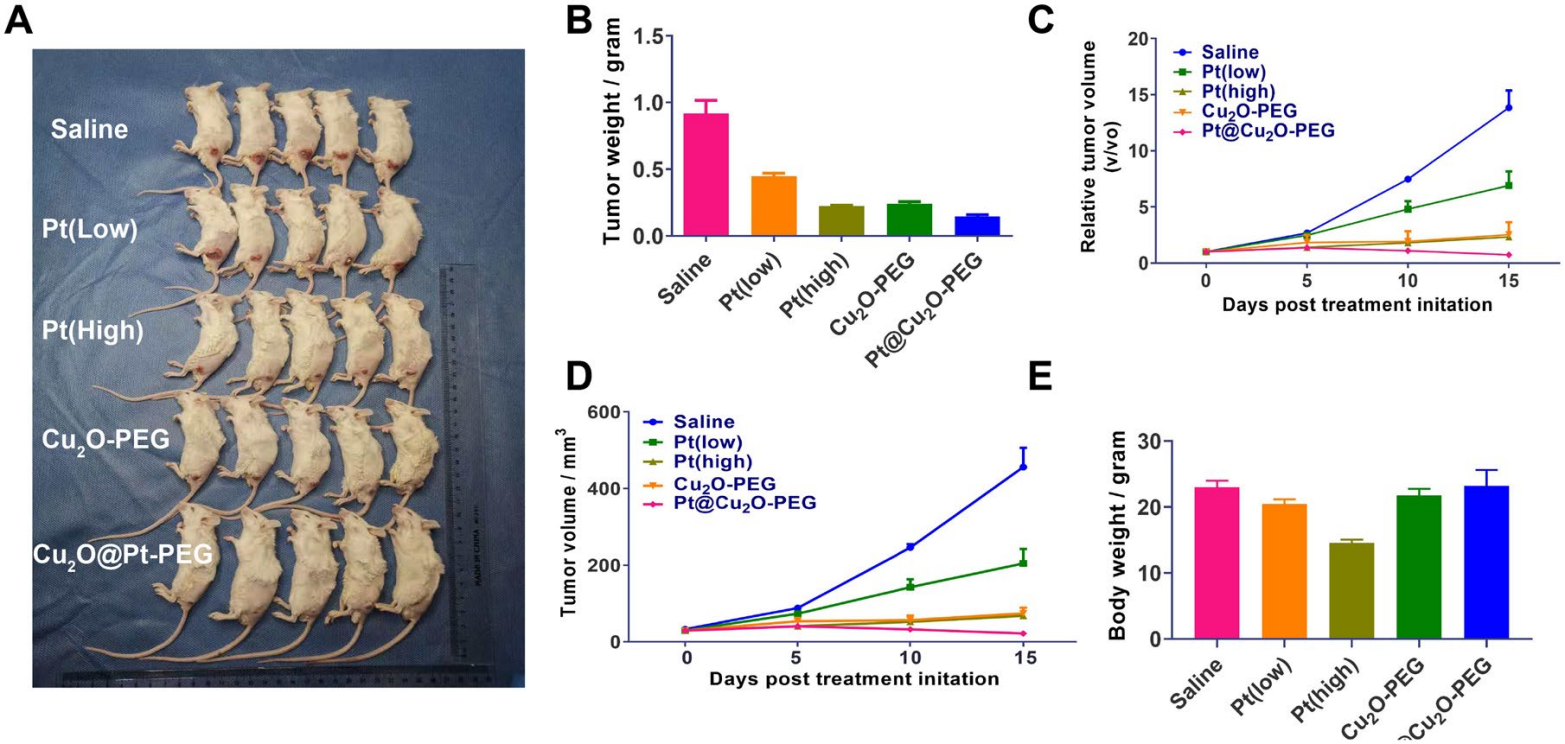
*In vivo* therapeutic properties of Cu_2_O@Pt-PEG NPs. A: Images of the primary tumors derived from mice with different treatments. B: Body weights following the recommended procedures. C: Tumor volumes and Relative tumor volumes (D) after the recommended remedies. E: Tumor weights after the indicated treatments.

### 3.5. Biosafety and metabolism study and anti-Tumor efficiency of Cu_2_O@Pt-PEG nanocrystals *in vivo*

To explore the biosafety and in vivo metabolism of inorganic nanomaterials, relevant experiment have been conducted. As demonstrated in Supplementary Figure S10, the Cu_2_O@Pt-PEG NPs are injected intravenously, it was primarily taken up by liver, spleen, kidney, and tumor, and they are gradually processed and eliminated by liver and kidney after 24 h. In addition, tissues stained with H&E were used to evaluate the biosafety of Cu_2_O@Pt-PEG NPs in the treatment of major organs, and no inflammation or damage was observed (Supplementary Figure S11).

We have confirmed that Cu_2_O@Pt-PEG can effectively exert anti-tumor effects *in vitro*, and then we further tested the anti-tumor effect of Cu_2_O@Pt-PEG on tumor-bearing mice, which were treated with Pt (low dose), Pt (high dose), Cu_2_O-PEG or Cu_2_O@Pt-PEG (with a low dose of Pt), and saline (negative control) every 2 days. The results of *in vivo* experiments showed that the tumor volumes and tumor weights in Pt (low dose), Pt (high dose), Cu_2_O-PEG and Cu_2_O@Pt-PEG (with low dose of Pt) groups did being significantly reduced, compared to those in saline group. Among all, Pt (low) showed a ∼50% inhibitory effect, which was much less than those in Pt (high) group with a ∼65% inhibitory response. To date, Cu_2_O-PEG also showed well anti-proliferation capacity (∼70%), whereas the combination effect of chem plus CDT resulted from Cu_2_O@Pt-PEG, showed most efficient anti-growth effect to tumor (Figure 5(A,C–E)). Apart from this, the safety of anticancer drugs is an important consideration in clinical research. To further evaluate their potential side effects, we tested the weight change of mice throughout the experiment, and the results showed that the weight changes of Cu_2_O-PEG and Cu_2_O@Pt-PEG groups of mice were not statistically different from control group. However, both Pt-treated groups showed significant decreased body weight, whereas Pt (high) demonstrated lower body weights compared to Pt (low). This phenomenon indicates that Pt chemo-agents showed dose-dependent side toxicity to mice, while Cu_2_O-PEG and Cu_2_O@Pt-PEG has no significant side effects (Figure 5(B)).

All results aforementioned implied that Cu_2_O@Pt-PEG had a remarkable antitumor effect and the laser could enhance this antitumor effect *in vivo.* In summary, Cu_2_O@Pt-PEG has good prospects in the treatment of cancer.

## 4. Conclusions

**Scheme 1.**
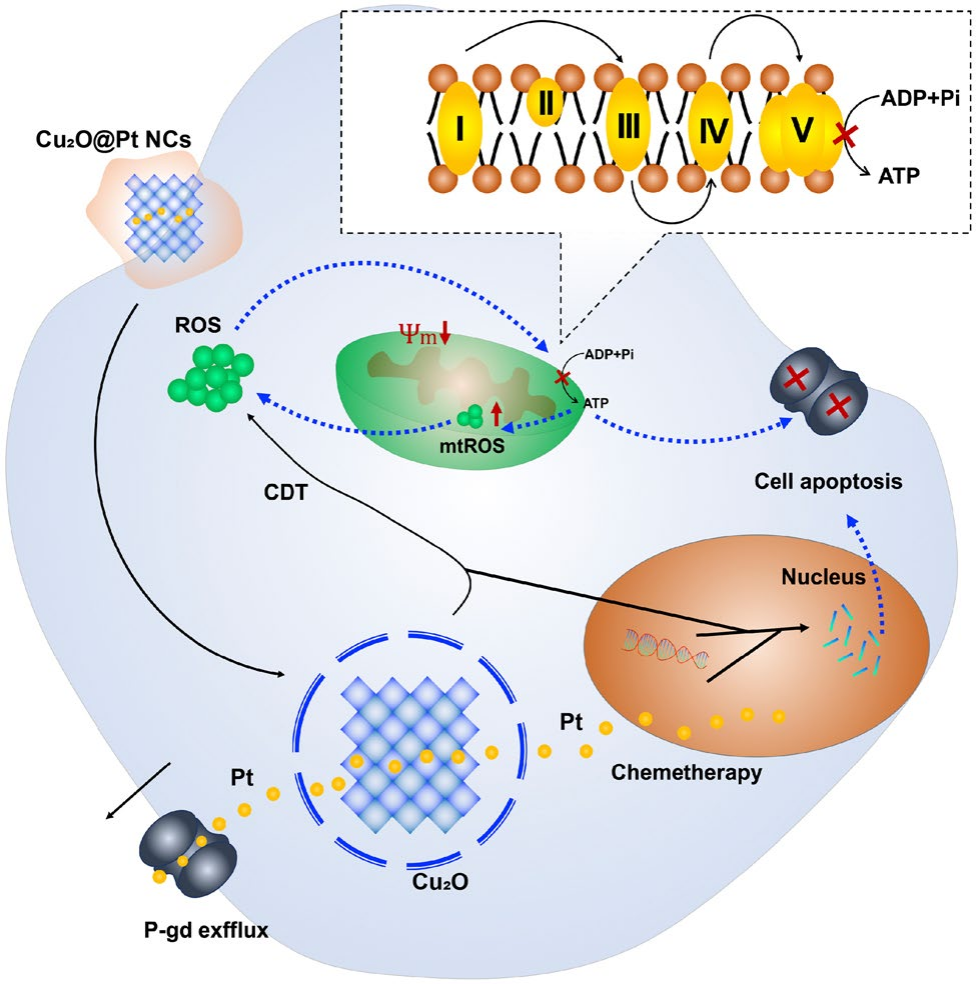
Schematic illustration of Cu2O@Pt-PEG nanosheets for overcome tumor drug resistance.

In conclusion, we developed novel nanocrystals (Cu_2_O@Pt NCs) to overcome drug resistance by reducing mitochondria-derived ATP through chemo/CDT in GI cancer. The Cu_2_O@Pt NCs were able to carry out CDT. Because of the oxidative stress induced by CDT, the Cu_2_O@Pt NCs were capable of elevating ROS levels and eliciting mitochondrial dysfunction and furthermore, the release of cisplatin further impaired mitochondrial function. What’s more, P-gp’s function could be inhibited by the reduction of mitochondria-derived ATP through chemo/CDT, which reduce cisplatin excretion and reverse drug resistance. *In vivo* results in tumor-bearing mice demonstrated that treatment with Cu_2_O@Pt NCs with CDT and chemotherapy could achieve the most effective antitumor therapeutic effect with the least amounts of adverse effects (Scheme 1).

## Supplementary Material

Supplemental Material

## Data Availability

The datasets used in the present study are available from the corresponding author upon reasonable request.

## References

[CIT0001] Adepu S, Ramakrishna S. (2021). Controlled drug delivery systems: current status and future directions. Molecules 26:5905. 34641447 10.3390/molecules26195905 PMC8512302

[CIT0002] Akahoshi K, Oya M, Koga T, Shiratsuchi Y. (2018). Current clinical management of gastrointestinal stromal tumor. World J Gastroenterol 24:2806–17. 30018476 10.3748/wjg.v24.i26.2806 PMC6048423

[CIT0003] Behranvand N, Nasri F, Zolfaghari ER, et al. (2022). Chemotherapy: a double-edged sword in cancer treatment. Cancer Immunol Immunother 71:507–26. 34355266 10.1007/s00262-021-03013-3 PMC10992618

[CIT0004] Bray F, Ferlay J, Soerjomataram I, et al. (2018). Global cancer statistics 2018: GLOBOCAN estimates of incidence and mortality worldwide for 36 cancers in 185 countries. CA Cancer J Clin 68:394–424. 30207593 10.3322/caac.21492

[CIT0005] Bregni G, Akin TT, Camera S, et al. (2020). Adjuvant chemotherapy for rectal cancer: Current evidence and recommendations for clinical practice. Cancer Treat Rev 83:101948. 31955069 10.1016/j.ctrv.2019.101948

[CIT0006] Carneiro BA, El-Deiry WS. (2020). Targeting apoptosis in cancer therapy. Nat Rev Clin Oncol 17:395–417. 32203277 10.1038/s41571-020-0341-y PMC8211386

[CIT0007] Chang M, Hou Z, Jin D, et al. (2020). Colorectal tumor microenvironment-activated bio-decomposable and metabolizable Cu_2_O@CaCO_3_ nanocomposites for synergistic oncotherapy. Adv Mater 32:e2004647. 32945002 10.1002/adma.202004647

[CIT0008] Chau LY, Qijin H, Ailin Q, et al. (2016). Platinum nanoparticles on reduced graphene oxide as peroxidase mimetics for the colorimetric detection of specific DNA sequence. J Mater Chem B 4:4076–83. 32264609 10.1039/c6tb00741d

[CIT0009] Chen X, Wang H, Huang Y, et al. (2021). Future perspectives of exosomes in peritoneal metastasis of gastric cancer. Front Oncol 2021;11. 10.3389/fonc.2021.684871 PMC8276633 34268118

[CIT0010] Dilruba S, Kalayda GV. (2016). Platinum-based drugs: past, present and future. Cancer Chemother Pharmacol 77:1103–24. 26886018 10.1007/s00280-016-2976-z

[CIT0011] Gao L, Song Y, Zhong J, et al. (2022). Biocompatible 2D Cu-TCPP nanosheets derived from Cu_2_O nanocubes as multifunctional nanoplatforms for combined anticancer therapy. ACS Biomater Sci Eng 8:1074–86. 35129963 10.1021/acsbiomaterials.1c01430

[CIT0012] Goos JACM, Cho A, Carter LM, et al. (2020). Delivery of polymeric nanostars for molecular imaging and endoradiotherapy through the enhanced permeability and retention (EPR) effect. Theranostics 10:567–84. 31903138 10.7150/thno.36777 PMC6929988

[CIT0013] Heng P. (2018). Controlled release drug delivery systems. Pharm Dev Technol 23:833. 30375914 10.1080/10837450.2018.1534376

[CIT0014] Jain KK. (2020). An overview of drug delivery systems. Methods Mol Biol 2059:1–54. 31435914 10.1007/978-1-4939-9798-5_1

[CIT0015] Johnstone TC, Wilson JJ, Lippard SJ. (2013). Monofunctional and higher-valent platinum anticancer agents. Inorg Chem 52:12234–49. 23738524 10.1021/ic400538c PMC3818431

[CIT0016] Kobayashi H, Watanabe R, Choyke PL. (2013). Improving conventional enhanced permeability and retention (EPR) effects; what is the appropriate target? Theranostics 4:81–9. 24396516 10.7150/thno.7193 PMC3881228

[CIT0017] Leopoldo M, Nardulli P, Contino M, et al. (2018). An updated patent review on P-glycoprotein inhibitors (2011-2018). Expert Opin Ther Pat 29:455–61. 10.1080/13543776.2019.1618273 31079547

[CIT0018] Li Q, Zhou Y, He W, et al. (2021). Platelet-armored nanoplatform to harmonize janus-faced IFN-gamma against tumor recurrence and metastasis. J Control Release 338:33–45. 34391837 10.1016/j.jconrel.2021.08.020

[CIT0019] Mollazadeh S, Sahebkar A, Hadizadeh F, et al. (2018). Structural and functional aspects of P-glycoprotein and its inhibitors. Life Sci 214:118–23. 30449449 10.1016/j.lfs.2018.10.048

[CIT0020] Rottenberg S, Disler C, Perego P. (2021). The rediscovery of platinum-based cancer therapy. Nat Rev Cancer 21:37–50. 33128031 10.1038/s41568-020-00308-y

[CIT0021] Sung H, Ferlay J, Siegel RL, et al. (2021). Global Cancer Statistics 2020: GLOBOCAN estimates of incidence and mortality worldwide for 36 cancers in 185 countries. CA Cancer J Clin 71:209–49. 33538338 10.3322/caac.21660

[CIT0022] Tang Z, Liu Y, He M, Bu W. (2019). Chemodynamic therapy: tumour microenvironment-mediated fenton and fenton-like reaction. Angew Chem Int Ed Engl 58:946–56. 30048028 10.1002/anie.201805664

[CIT0023] Uggeri J, Gatti R, Belletti S, et al. (2004). Calcein-AM is a detector of intracellular oxidative activity. Histochem Cell Biol 122:499–505. 15503120 10.1007/s00418-004-0712-y

[CIT0024] Unsoy G, Gunduz U. (2018). Smart drug delivery systems in cancer therapy. Curr Drug Targets 19:202–12. 27033191 10.2174/1389450117666160401124624

[CIT0025] Venerito M, Link A, Rokkas T, Malfertheiner P. (2016). Gastric cancer – clinical and epidemiological aspects. Helicobacter 21:39–44. 27531538 10.1111/hel.12339

[CIT0026] Waghray D, Zhang Q. (2018). Inhibit or evade multidrug resistance P-glycoprotein in cancer treatment. J Med Chem 61:5108–21. 29251920 10.1021/acs.jmedchem.7b01457 PMC6281405

[CIT0027] Wu H, Chen F, You C, et al. (2020). Smart porous core-shell cuprous oxide nanocatalyst with high biocompatibility for acid-triggered chemo/chemodynamic synergistic therapy. Small 16:e2001805. 33079449 10.1002/smll.202001805

[CIT0028] Yang B, Chen Y, Shi J. (2019). Reactive oxygen species (ROS)-based nanomedicine. Chem Rev 119:4881–985. 30973011 10.1021/acs.chemrev.8b00626

[CIT0029] Yang Y, Yu Y, Chen H, et al. (2020). Illuminating platinum transportation while maximizing therapeutic efficacy by gold nanoclusters via simultaneous near-infrared-I/II imaging and glutathione scavenging. ACS Nano 14:13536–47. 32924505 10.1021/acsnano.0c05541

[CIT0030] Zhang M, Qin X, Zhao Z, et al. (2022). A self-amplifying nanodrug to manipulate the Janus-faced nature of ferroptosis for tumor therapy. Nanoscale Horiz 7:198–210. 35023537 10.1039/d1nh00506e

