# Supplementary material for: Cuprous oxide-based nanocrystals with combined chemo/chemodynamic therapy to increase tumor drug sensitivity by reducing mitochondria-derived adenosine-triphosphate
Source: Drug Deliv. 2022 Sep 25;29(1):3134–41. doi: 10.1080/10717544.2022.2121450 (PMC9518603; doi:10.1080/10717544.2022.2121450)
Supplement: Supplemental Material [file IDRD_A_2121450_SM7162.docx]

Supporting Information

Cuprous oxide (Cu_2_O)-based nanocrystals with combined chemo/chemodynamic therapy to overcome tumor drug resistance by reducing mitochondria-derived adenosine-triphosphate (ATP)


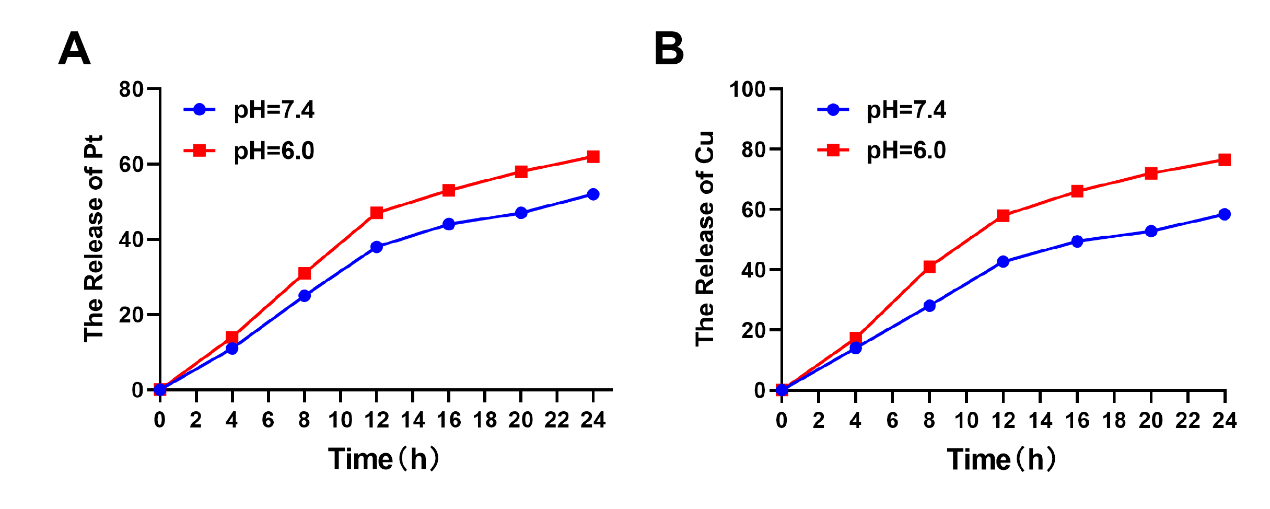


**Figure S1.** Time-dependent Pt (A)and Cu (B) release from the Cu_2_O@Pt NCs with low pH solution.


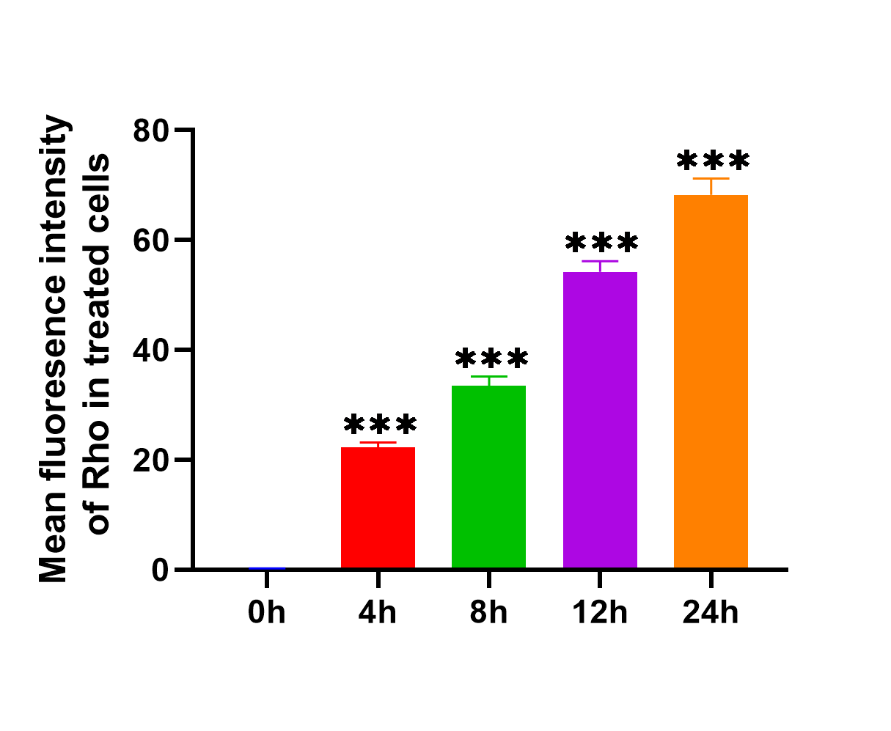


**Figure S2.** Statistical analysis of CLSM images of AGS cells treated with Cu_2_O@Pt-PEG NCs for 0-24 h. Data are shown as mean±SD, n=3.

*** indicates *P*<0.001


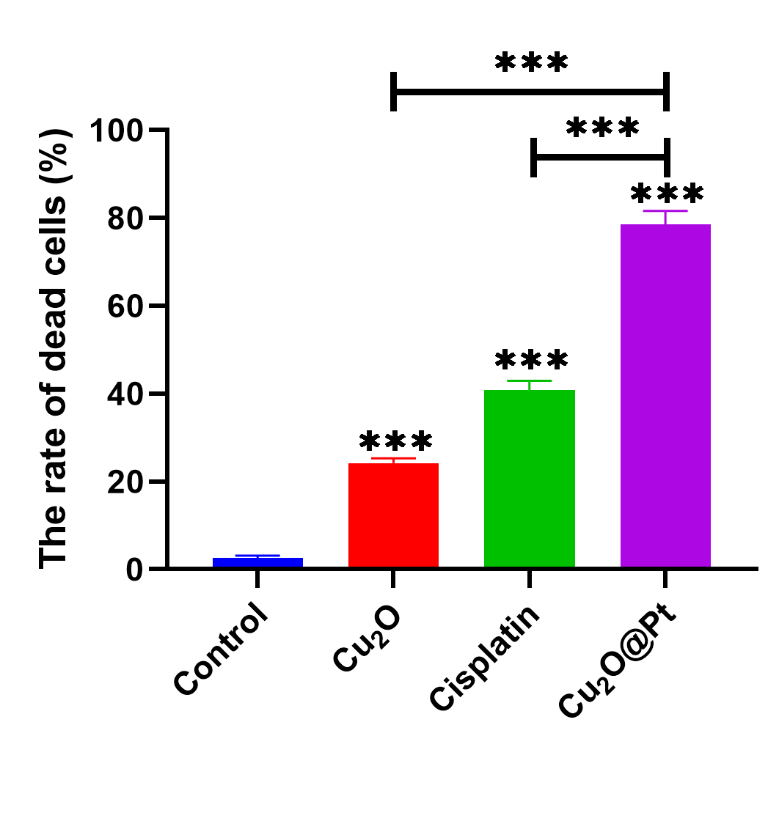


**Figure S3.** Statistical analysis of the rate of dead cells in AGS cells after different treatments. Data are shown as mean±SD, n=3. *** indicates *P*<0.001.


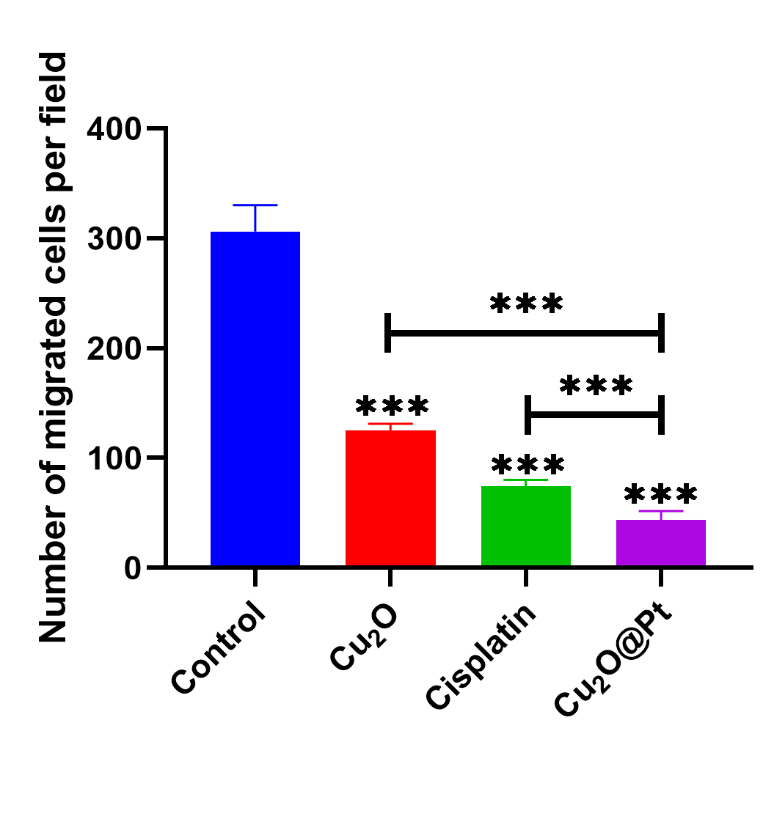


**Figure S4.** Statistical analysis of Transwell assays for AGS cells. Data are shown as mean±SD, n=3. *** indicates *P*<0.001


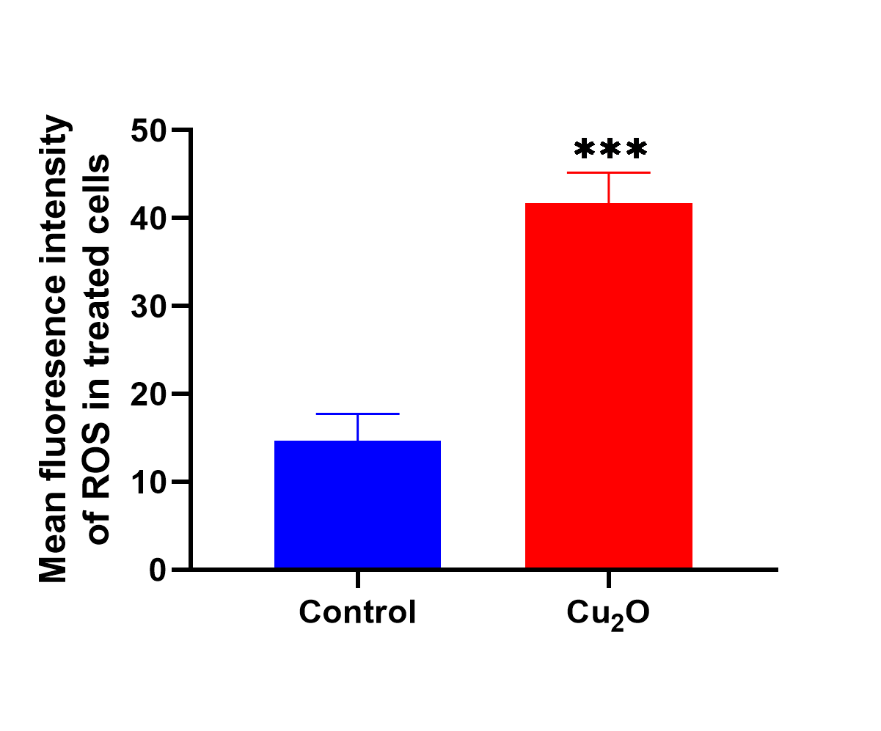


**Figure S5.** Statistical analysis of the mean fluorescence intensity of ROS in AGS cells after treatments. Data are shown as mean±SD, n=3.

*** indicates *P*<0.001


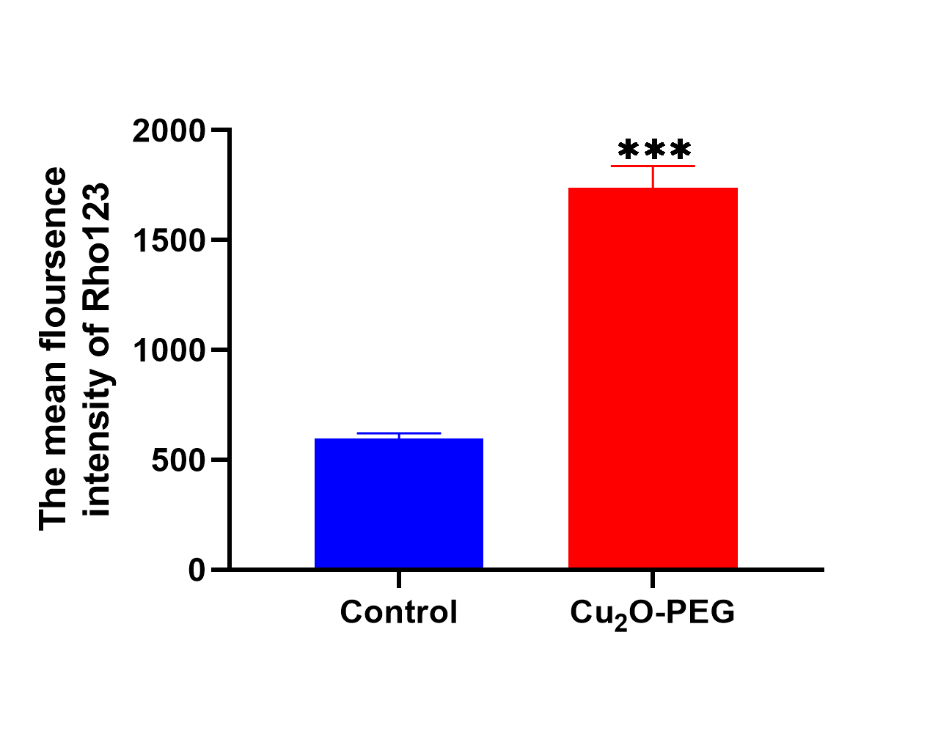


**Figure S6.** Statistical analysis of the mean fluoresence intensity of Rho123 in AGS cells after treatments. Data are shown as mean±SD, n=3.

*** indicates *P*<0.001.


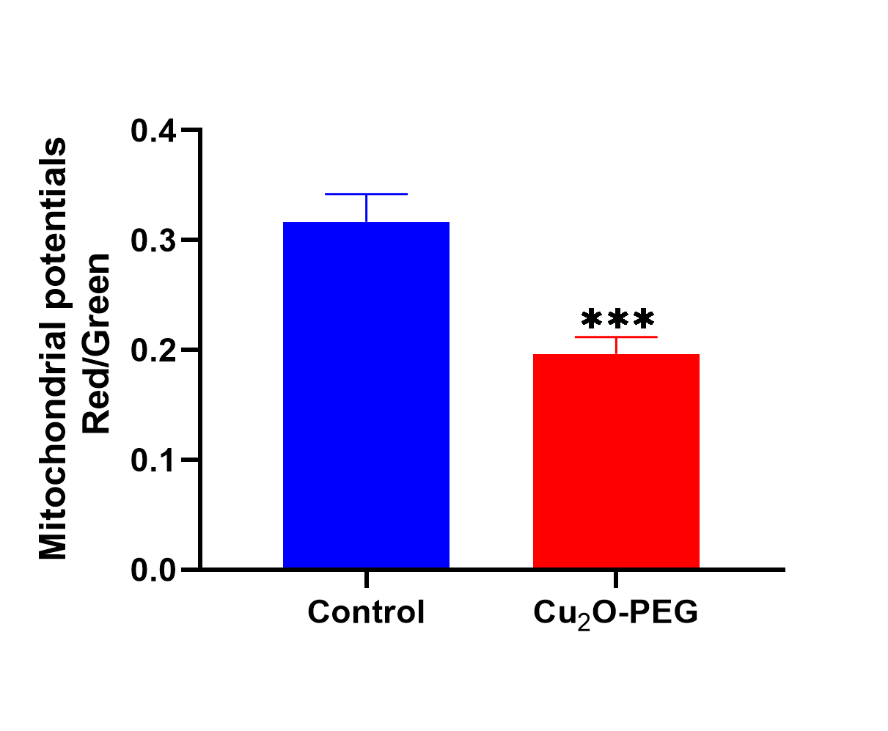


**Figure S7.** Statistical analysis of the mean fluoresence intensity of JC-1 in AGS cells after different treatments. Data are shown as mean±SD, n=3. *** indicates *P*<0.001.


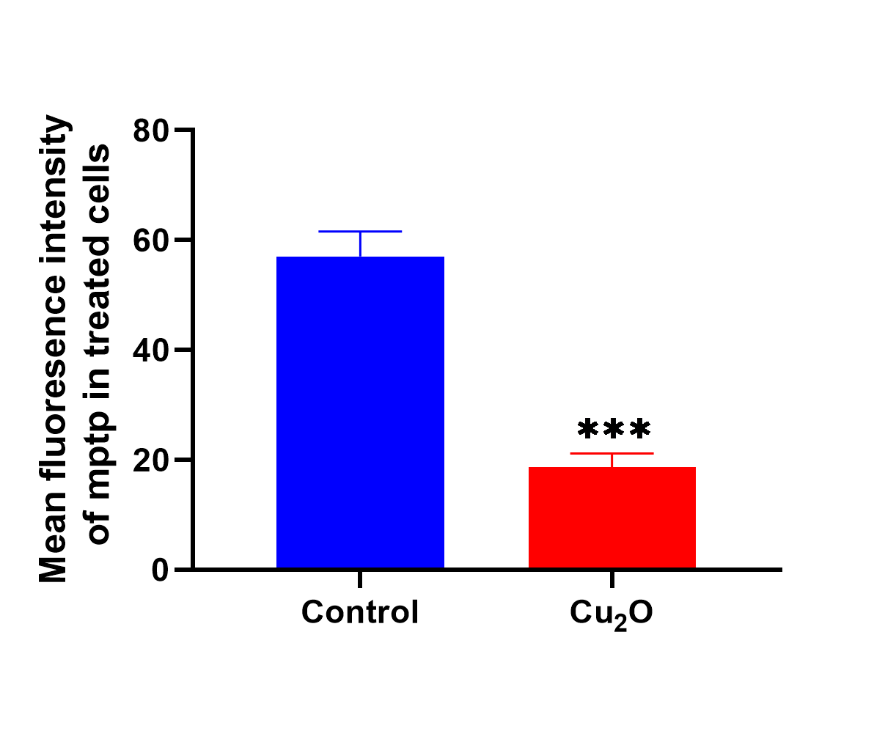


**Figure S8.** Statistical analysis of the mean fluoresence intensity of mPTP in AGS cells after treatments. Data are shown as mean±SD, n=3.

*** indicates *P*<0.001.


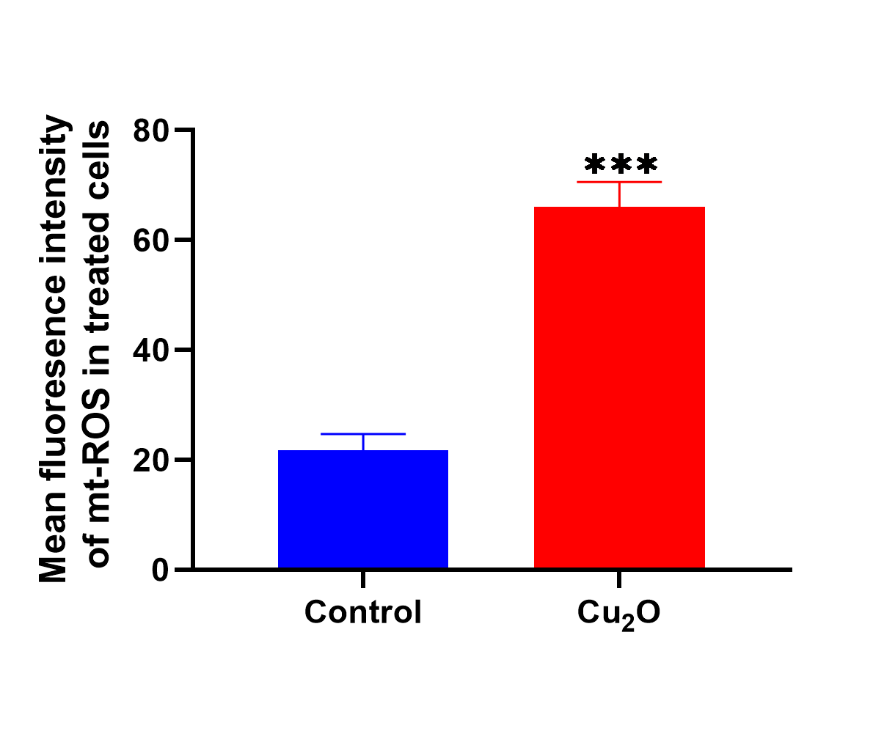


**Figure S9.** Statistical analysis of the mean fluoresence intensity of mt-ROS in AGS cells after treatments. Data are shown as mean±SD, n=3.

*** indicates *P*<0.001.


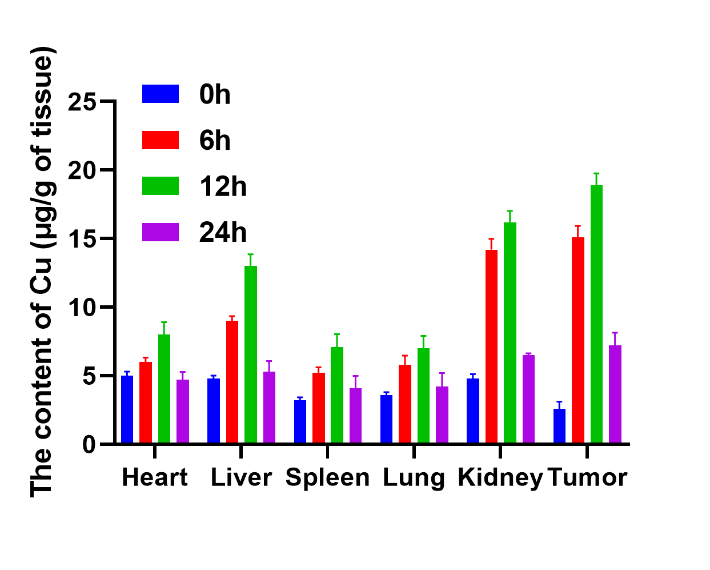


**Figure S10** Injection of Cu_2_O@Pt NCs into major organs (heart, liver, spleen, lung, kidney) and tumors detected by ICP-MS after i.v. injection (dose = 2.5 mg/kg Cu, 120 μL) for different time (n=3).


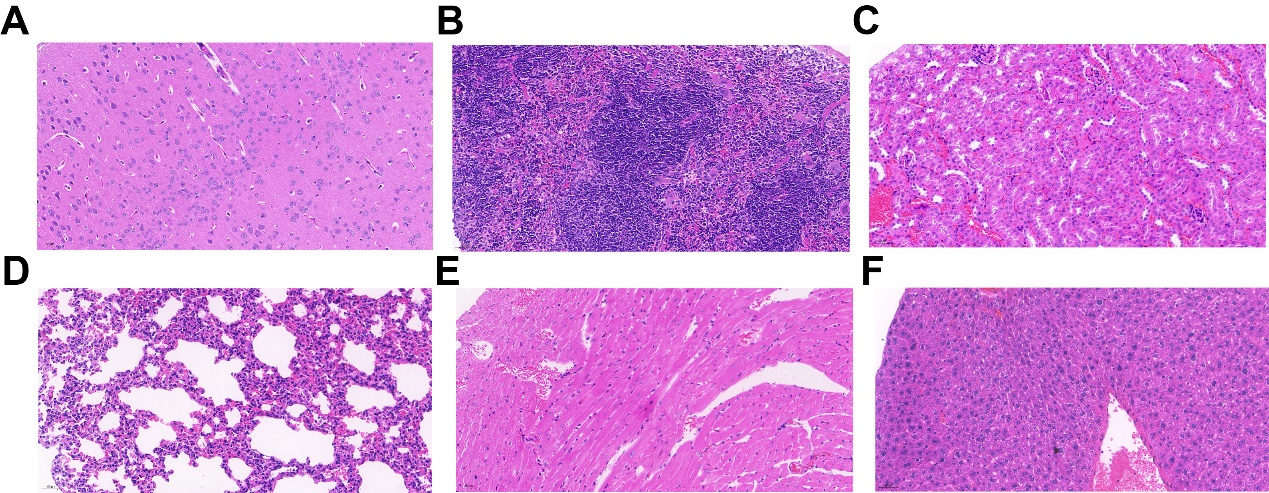


**Figure S11** H&E staining images of major organs (Brains, spleens, kidneys, lungs, hearts, and livers) of bearing MFC tumor mice in treatment of injection of Cu_2_O@Pt NCs.
